# 1*H*,3*H*-Imidazolium (*R*,*S*)-camphor-10-sulfonate

**DOI:** 10.1107/S1600536808043742

**Published:** 2009-01-08

**Authors:** Mohd Basyaruddin Abdul Rahman, Emmy Maryati Omar, Shie Ling Ng, Reza Kia, Hoong-Kun Fun

**Affiliations:** a Department of Chemistry, Faculty of Science, Universiti Putra Malaysia, 43400 UPM Serdang, Selangor, Malaysia; b Queen’s University Ionic Liquid Laboratory (QUILL), David Keir Building, Stransmillis Road, BT9 5AG Belfast, Northern Ireland, United Kingdom; c X-ray Crystallography Unit, School of Physics, Universiti Sains Malaysia, 11800 USM, Penang, Malaysia

## Abstract

The title compound, C_3_H_5_N_2_^+^·C_10_H_15_O_4_S^−^, comprises two crystallographically independent ion pairs (*A* and *B*) in the asymmetric unit with slightly different conformations due to the disordered methyl groups in the anion of molecule *A*. Two intra­molecular C—H⋯O hydrogen bonds generate *S*(6) ring motifs. In mol­ecule *A*, the methyl groups are disordered over two sets of positions with a site-ocuppancy ratio of 0.547 (9):0.453 (9). Extensive inter­molecular N—H⋯O and C—H⋯O hydrogen-bonding inter­actions occur in the crystal structure which link the mol­ecules into a two-dimensional network parallel to the (100) plane.

## Related literature

For hydrogen-bond motifs, see: Bernstein *et al.* (1995 ▶). For bond-length data, see: Allen *et al.* (1987 ▶). For general background, see: Fukumoto *et al.* (2005 ▶); Jeremić *et al.* (2008 ▶).
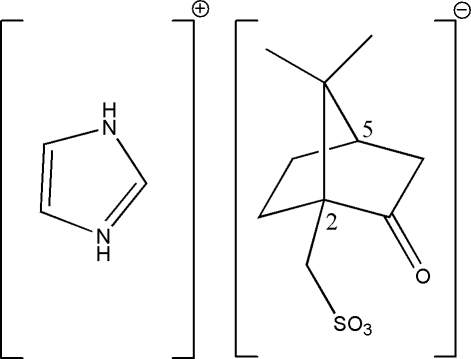


## Experimental

### 

#### Crystal data

C_3_H_5_N_2_^+^·C_10_H_15_O_4_S^−^
*M* *_r_* = 300.37
Monoclinic, 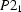

*a* = 9.1362 (2) Å
*b* = 12.0126 (2) Å
*c* = 13.2526 (3) Å
β = 90.757 (1)°
*V* = 1454.34 (5) Å^3^
*Z* = 4
Mo *K*α radiation
μ = 0.24 mm^−1^
*T* = 100.0 (1) K
0.46 × 0.45 × 0.19 mm

#### Data collection

Bruker SMART APEXII CCD area-detector diffractometer
Absorption correction: multi-scan (**SADABS**; Bruker, 2005 ▶) *T*_min_ = 0.900, *T*_max_ = 0.956
25973 measured reflections
9954 independent reflections
9652 reflections with *I* > 2σ(*I*)
*R*_int_ = 0.022

#### Refinement

*R*[*F* ^2^ > 2σ(*F* ^2^)] = 0.031
*wR*(*F* ^2^) = 0.083
*S* = 1.07
9954 reflections
355 parameters
1 restraint
H-atom parameters constrained
Δρ_max_ = 0.53 e Å^−3^
Δρ_min_ = −0.34 e Å^−3^
Absolute structure: Flack (1983 ▶), 4199 Friedel pairs
Flack parameter: 0.01 (3)

### 

Data collection: *APEX2* (Bruker, 2005 ▶); cell refinement: *SAINT* (Bruker, 2005 ▶); data reduction: *SAINT*; program(s) used to solve structure: *SHELXTL* (Sheldrick, 2008 ▶); program(s) used to refine structure: *SHELXTL*; molecular graphics: *SHELXTL*; software used to prepare material for publication: *SHELXTL* and *PLATON* (Spek, 2003 ▶).

## Supplementary Material

Crystal structure: contains datablocks global, I. DOI: 10.1107/S1600536808043742/ww2136sup1.cif

Structure factors: contains datablocks I. DOI: 10.1107/S1600536808043742/ww2136Isup2.hkl

Additional supplementary materials:  crystallographic information; 3D view; checkCIF report

## Figures and Tables

**Table 1:** Hydrogen-bond geometry (Å, °)

*D*—H⋯*A*	*D*—H	H⋯*A*	*D*⋯*A*	*D*—H⋯*A*
N1*A*—H1*AC*⋯O2*B*^i^	0.86	2.46	2.9479 (14)	116
N1*A*—H1*AC*⋯O3*A*^ii^	0.86	2.00	2.8293 (14)	161
N2*A*—H2*AA*⋯O1*B*^iii^	0.86	1.89	2.7235 (14)	164
N1*B*—H1*BC*⋯O1*A*^i^	0.86	1.88	2.7231 (14)	165
N2*B*—H2*BA*⋯O2*B*^iii^	0.86	1.97	2.7412 (14)	148
N2*B*—H2*BA*⋯O3*A*^iv^	0.86	2.43	3.0111 (14)	125
C7*A*—H7*AB*⋯O1*A*	0.97	2.53	3.0257 (19)	111
C11*A*—H11*A*⋯O2*B*^i^	0.93	2.49	2.9716 (16)	113
C11*A*—H11*A*⋯O3*B*^i^	0.93	2.35	3.2648 (15)	170
C11*B*—H11*B*⋯O2*A*^iv^	0.93	2.33	3.2103 (16)	159
C9*A*—H9*AC*⋯O2*A*^i^	0.96	2.59	3.469 (3)	152
C12*B*—H12*B*⋯O3*B*^i^	0.93	2.39	2.9950 (15)	122
C12*B*—H12*B*⋯O4*A*^i^	0.93	2.49	3.0155 (16)	116
C13*A*—H13*A*⋯O2*A*^iii^	0.93	2.44	3.0167 (16)	120
C13*A*—H13*A*⋯O4*A*^iii^	0.93	2.39	3.2591 (17)	155
C13*B*—H13*B*⋯O1*B*^iii^	0.93	2.58	3.2720 (16)	131
C7*B*—H7*BA*⋯O2*B*	0.97	2.47	3.0613 (16)	119
C5*A*—H5*AA*⋯*Cg*1	0.98	2.83	3.5459 (5)	131

Symmetry codes: (i) 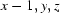; (ii) 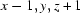; (iii) 
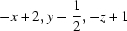
; (iv) 
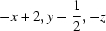
.

**Fig. 1.**
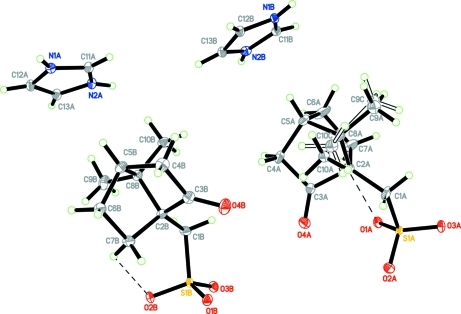
The molecular structure of (I) with atom labels and 30% probability ellipsoids for non-H atoms. Intramolecular interactions are shown as dashed lines. Open bonds show the minor component.

**Fig. 2.**
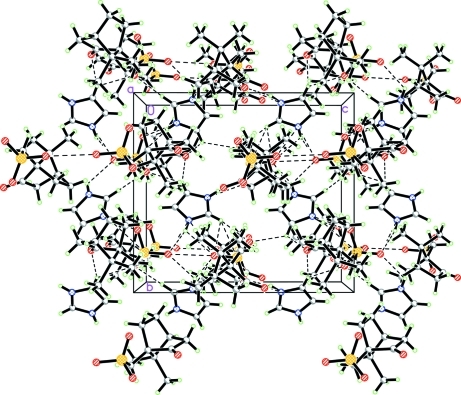
The crystal packing of (I), viewed down the a-axis showing infinite 1-D chains along the b and c-axes of the unit cell. Intermolecular interactions are shown as dashed lines. Only the major component of molecule A is shown.

## supplementary crystallographic information

### Comment

The title compound, (I, Fig. 1), is based on alkyl-imidazole with plant acid as anion (halogen-free). Crystallization of the 1,3-dihydrogenimidazolium camphor-10 -sulfonate having a sulfonate ion as a counter-ion was achieved by a slow evaporation of methanol at ambient temperature. Camphorsulfonate anion was selected due to their low toxicity for biocatalysis applications (Jeremić *et al.*, 2008). The title compound has strong ion-ion interactions between cations and anions, which was attributed to an increase in the van der Waals attraction between the alkyl groups (Fukumoto *et al.*, 2005).

In the title compound (I, Fig. 1), the bond lengths (Allen *et al.*, 1987) and angles are within the normal ranges. There are two intramolecular C—H···O interactions generating six-membered rings with *S(6)* ring motifs (Bernstein *et al.*, 1995). In the molecule A, the methyl groups are disordered over two positions and refined isotropically with site-ocuppancy ratio of 0.547 (9)/0.453 (9). In the crystal structure, molecules are linked together into 1-D infinite chains along the *b* axis, and are also linked into 1-D infinite chains along the *c* axis, thus forming a 2-D network which is parallel to the (100)-plane. The crystal structure is stabilized by intermolecular N—H···O (*x* 6) and C—H···O (*x* 9) hydrogen bonds, and weak intermlecular C—H···π interactions (*Cg1* is the centroid of the N1B/C11B/N2B/C13B/C12B ring).

### Experimental

(*R*)-(-)-Camphor-10-sulfonic acid (0.05 mol, 7.504 g) was added to imidazole (0.05 mol, 3.404 g) which was first dissolved in 20 ml of methanol. The mixture was stirred (150 rpm) for 2 h at room temperature and the excess methanol was removed *in vacuo* at 343 K. The final product was obtained as a white solid with 97% yield. It was then dried under high vacuum for 2 days. m.p. 559.77 K. Single Crystals suitable for *X*-ray analysis was obtained from methanol. Anal. Calc.: C, 51.98; H, 6.71; N, 9.33; O, 21.31; S, 10.67. Found: C, 51.97; H, 6.77; N, 9.11; O, 21.38; S, 10.77%.

### Refinement

All the hydrogen atoms were positioned geometrically and constrained to ride with the parent atoms with *U*_iso_(H) = 1.2 or 1.5 *U*_eq_ (O or N). In molecule A, the methyl groups are disordered over two positions and refined isotropically with a site-occupancy ratio of 0.547 (9)/0.453 (9), because anisotropic refinement casues non-positive definiteness for these atoms. Sufficient anomalous scattering due to the presence of S atoms gave the correct value of the Flack parameter which lead to the correct absolute configuration given in Fig. 1. Floating origin restraint was applied automatically by *SHELXL* program for this chiral space group, P2_1_.

### Crystal data

**Table:**

C_3_H_5_N_2_^+^·C_10_H_15_O_4_S^−^	*F*(000) = 640
*M**_r_* = 300.37	*D*_x_ = 1.372 Mg m^−^^3^
Monoclinic, *P*2_1_	Mo *K*α radiation, λ = 0.71073 Å
Hall symbol: P 2yb	Cell parameters from 9809 reflections
*a* = 9.1362 (2) Å	θ = 2.3–38.9°
*b* = 12.0126 (2) Å	µ = 0.24 mm^−^^1^
*c* = 13.2526 (3) Å	*T* = 100 K
β = 90.757 (1)°	Block, colourless
*V* = 1454.34 (5) Å^3^	0.46 × 0.45 × 0.19 mm
*Z* = 4	

### Data collection

**Table:**

Bruker SMART APEXII CCD area-detector diffractometer	9954 independent reflections
Radiation source: fine-focus sealed tube	9652 reflections with *I* > 2σ(*I*)
graphite	*R*_int_ = 0.022
φ and ω scans	θ_max_ = 32.5°, θ_min_ = 1.5°
Absorption correction: multi-scan (*SADABS*; Bruker, 2005)	*h* = −13→13
*T*_min_ = 0.900, *T*_max_ = 0.956	*k* = −17→18
25973 measured reflections	*l* = −17→20

### Refinement

**Table:**

Refinement on *F*^2^	Secondary atom site location: difference Fourier map
Least-squares matrix: full	Hydrogen site location: inferred from neighbouring sites
*R*[*F*^2^ > 2σ(*F*^2^)] = 0.031	H-atom parameters constrained
*wR*(*F*^2^) = 0.083	*w* = 1/[σ^2^(*F*_o_^2^) + (0.047*P*)^2^ + 0.3247*P*] where *P* = (*F*_o_^2^ + 2*F*_c_^2^)/3
*S* = 1.07	(Δ/σ)_max_ = 0.002
9954 reflections	Δρ_max_ = 0.53 e Å^−^^3^
355 parameters	Δρ_min_ = −0.34 e Å^−^^3^
1 restraint	Absolute structure: Flack (1983), 4199 Friedel pairs
Primary atom site location: structure-invariant direct methods	Flack parameter: 0.01 (3)

### Special details

**Table:**

Experimental. The low-temperature data was collected with the Oxford Cyrosystem Cobra low-temperature attachment.
Geometry. All e.s.d.'s (except the e.s.d. in the dihedral angle between two l.s. planes) are estimated using the full covariance matrix. The cell e.s.d.'s are taken into account individually in the estimation of e.s.d.'s in distances, angles and torsion angles; correlations between e.s.d.'s in cell parameters are only used when they are defined by crystal symmetry. An approximate (isotropic) treatment of cell e.s.d.'s is used for estimating e.s.d.'s involving l.s. planes.
Refinement. Refinement of *F*^2^ against ALL reflections. The weighted *R*-factor *wR* and goodness of fit *S* are based on *F*^2^, conventional *R*-factors *R* are based on *F*, with *F* set to zero for negative *F*^2^. The threshold expression of *F*^2^ > σ(*F*^2^) is used only for calculating *R*-factors(gt) *etc*. and is not relevant to the choice of reflections for refinement. *R*-factors based on *F*^2^ are statistically about twice as large as those based on *F*, and *R*- factors based on ALL data will be even larger.

### Fractional atomic coordinates and isotropic or equivalent isotropic displacement parameters (Å^2^)

**Table:**

	*x*	*y*	*z*	*U*_iso_*/*U*_eq_	Occ. (<1)
S1A	1.27663 (3)	0.30766 (2)	−0.033021 (19)	0.01112 (5)	
O1A	1.29170 (10)	0.19188 (8)	0.00012 (7)	0.01629 (16)	
O2A	1.37452 (10)	0.38323 (9)	0.02074 (7)	0.01975 (18)	
O3A	1.28934 (9)	0.31774 (8)	−0.14282 (6)	0.01632 (16)	
O4A	1.17524 (10)	0.29350 (10)	0.20717 (7)	0.0227 (2)	
C1A	1.09258 (13)	0.35344 (13)	−0.00674 (10)	0.0230 (3)	
H1AA	1.0990	0.4290	0.0189	0.028*	
H1AB	1.0396	0.3569	−0.0706	0.028*	
C2A	1.00007 (12)	0.28760 (11)	0.06562 (9)	0.0161 (2)	
C3A	1.05299 (13)	0.27735 (10)	0.17433 (9)	0.0146 (2)	
C4A	0.91989 (14)	0.24497 (12)	0.23603 (10)	0.0206 (2)	
H4AA	0.8990	0.3005	0.2870	0.025*	
H4AB	0.9338	0.1733	0.2684	0.025*	
C5A	0.79837 (14)	0.24006 (14)	0.15568 (11)	0.0238 (3)	
H5AA	0.6991	0.2465	0.1822	0.029*	
C6A	0.8249 (2)	0.13498 (19)	0.09277 (16)	0.0449 (5)	
H6AA	0.7441	0.1211	0.0463	0.054*	
H6AB	0.8390	0.0703	0.1356	0.054*	
C7A	0.96719 (18)	0.16430 (16)	0.03538 (13)	0.0334 (4)	
H7AA	0.9522	0.1576	−0.0370	0.040*	
H7AB	1.0470	0.1158	0.0560	0.040*	
C8A	0.84295 (13)	0.33538 (17)	0.08391 (10)	0.0290 (3)	
C9A	0.7508 (3)	0.3652 (4)	−0.0072 (2)	0.0259 (7)*	0.547 (9)
H9AA	0.7369	0.3004	−0.0486	0.039*	0.547 (9)
H9AB	0.7993	0.4219	−0.0452	0.039*	0.547 (9)
H9AC	0.6574	0.3923	0.0143	0.039*	0.547 (9)
C9C	0.7443 (3)	0.3212 (4)	−0.0153 (2)	0.0206 (8)*	0.453 (9)
H9CA	0.7518	0.2461	−0.0395	0.031*	0.453 (9)
H9CB	0.7775	0.3717	−0.0663	0.031*	0.453 (9)
H9CC	0.6441	0.3374	0.0001	0.031*	0.453 (9)
C10A	0.8557 (3)	0.4563 (2)	0.1473 (2)	0.0172 (6)*	0.547 (9)
H10A	0.9196	0.4470	0.2047	0.026*	0.547 (9)
H10B	0.7604	0.4783	0.1694	0.026*	0.547 (9)
H10C	0.8945	0.5127	0.1038	0.026*	0.547 (9)
C10C	0.8303 (4)	0.4391 (3)	0.1224 (3)	0.0212 (8)*	0.453 (9)
H10D	0.7510	0.4773	0.0890	0.032*	0.453 (9)
H10E	0.9197	0.4793	0.1123	0.032*	0.453 (9)
H10F	0.8110	0.4344	0.1934	0.032*	0.453 (9)
N1A	0.44499 (11)	0.14307 (9)	0.76374 (8)	0.01573 (18)*	
H1AC	0.3885	0.1977	0.7787	0.019*	
N2A	0.56872 (12)	0.02395 (9)	0.67786 (8)	0.01563 (18)	
H2AA	0.6054	−0.0114	0.6278	0.019*	
C11A	0.47931 (13)	0.11135 (11)	0.67088 (9)	0.0155 (2)	
H11A	0.4466	0.1445	0.6113	0.019*	
C12A	0.51470 (14)	0.07405 (12)	0.83219 (9)	0.0176 (2)	
H12A	0.5091	0.0780	0.9021	0.021*	
C13A	0.59299 (14)	−0.00070 (11)	0.77835 (10)	0.0177 (2)	
H13A	0.6516	−0.0575	0.8042	0.021*	
S1B	1.29554 (3)	0.33636 (2)	0.532398 (19)	0.01117 (5)	
O1B	1.30840 (10)	0.45125 (8)	0.49628 (7)	0.01658 (16)	
O2B	1.32660 (9)	0.32743 (8)	0.64062 (6)	0.01474 (15)	
O3B	1.37993 (10)	0.25718 (8)	0.47370 (7)	0.01687 (17)	
O4B	0.95635 (13)	0.47418 (10)	0.40752 (8)	0.0267 (2)	
C1B	1.10979 (12)	0.29462 (11)	0.51462 (9)	0.0165 (2)	
H1BA	1.0902	0.2916	0.4426	0.020*	
H1BB	1.1005	0.2193	0.5402	0.020*	
C2B	0.98994 (12)	0.36491 (10)	0.56233 (9)	0.0141 (2)	
C3B	0.91119 (14)	0.44242 (11)	0.48786 (10)	0.0186 (2)	
C4B	0.76277 (16)	0.46991 (13)	0.53347 (11)	0.0233 (3)	
H4BA	0.6828	0.4448	0.4903	0.028*	
H4BB	0.7526	0.5491	0.5456	0.028*	
C5B	0.76907 (13)	0.40436 (11)	0.63263 (10)	0.0185 (2)	
H5BA	0.6734	0.3897	0.6625	0.022*	
C6B	0.87688 (15)	0.46647 (12)	0.70195 (11)	0.0217 (2)	
H6BA	0.8732	0.4381	0.7704	0.026*	
H6BB	0.8563	0.5457	0.7026	0.026*	
C7B	1.02824 (14)	0.44215 (12)	0.65360 (11)	0.0199 (2)	
H7BA	1.0933	0.4049	0.7012	0.024*	
H7BB	1.0743	0.5104	0.6311	0.024*	
C8B	0.85559 (12)	0.29851 (11)	0.60317 (9)	0.0154 (2)	
C9B	0.89102 (17)	0.22292 (13)	0.69361 (11)	0.0244 (3)	
H9BA	0.8036	0.1854	0.7143	0.037*	
H9BB	0.9285	0.2672	0.7485	0.037*	
H9BC	0.9631	0.1690	0.6746	0.037*	
C10B	0.78064 (14)	0.22785 (12)	0.52073 (11)	0.0207 (2)	
H10G	0.6992	0.1888	0.5490	0.031*	
H10H	0.8494	0.1752	0.4945	0.031*	
H10I	0.7465	0.2755	0.4672	0.031*	
N1B	0.42936 (11)	0.12226 (9)	0.17235 (8)	0.01373 (17)	
H1BC	0.3802	0.1537	0.1244	0.016*	
N2B	0.56682 (11)	0.00652 (9)	0.25337 (9)	0.01636 (19)	
H2BA	0.6221	−0.0496	0.2668	0.020*	
C11B	0.50552 (13)	0.02832 (11)	0.16403 (9)	0.0153 (2)	
H11B	0.5143	−0.0145	0.1060	0.018*	
C12B	0.44173 (13)	0.16124 (11)	0.27007 (9)	0.0154 (2)	
H12B	0.3992	0.2253	0.2960	0.018*	
C13B	0.52797 (14)	0.08788 (11)	0.32086 (9)	0.0171 (2)	
H13B	0.5556	0.0918	0.3886	0.021*	

### Atomic displacement parameters (Å^2^)

**Table:**

	*U*^11^	*U*^22^	*U*^33^	*U*^12^	*U*^13^	*U*^23^
S1A	0.01090 (10)	0.01332 (11)	0.00917 (10)	0.00054 (8)	0.00172 (8)	0.00011 (8)
O1A	0.0197 (4)	0.0149 (4)	0.0142 (4)	0.0028 (3)	−0.0013 (3)	0.0028 (3)
O2A	0.0213 (4)	0.0211 (5)	0.0168 (4)	−0.0046 (3)	−0.0011 (3)	−0.0040 (3)
O3A	0.0219 (4)	0.0171 (4)	0.0101 (3)	0.0017 (3)	0.0030 (3)	0.0002 (3)
O4A	0.0188 (4)	0.0302 (6)	0.0189 (4)	0.0012 (4)	−0.0020 (3)	−0.0005 (4)
C1A	0.0152 (5)	0.0307 (7)	0.0232 (6)	0.0082 (5)	0.0075 (4)	0.0153 (5)
C2A	0.0115 (4)	0.0247 (6)	0.0120 (5)	0.0004 (4)	0.0019 (3)	0.0015 (4)
C3A	0.0164 (5)	0.0129 (5)	0.0146 (5)	0.0023 (4)	0.0019 (4)	0.0019 (4)
C4A	0.0213 (5)	0.0236 (6)	0.0171 (5)	0.0022 (5)	0.0067 (4)	0.0062 (5)
C5A	0.0161 (5)	0.0345 (8)	0.0211 (6)	−0.0054 (5)	0.0068 (4)	−0.0012 (5)
C6A	0.0342 (8)	0.0530 (12)	0.0480 (10)	−0.0286 (8)	0.0241 (8)	−0.0278 (9)
C7A	0.0280 (7)	0.0392 (9)	0.0333 (8)	−0.0180 (6)	0.0153 (6)	−0.0200 (7)
C8A	0.0121 (5)	0.0538 (10)	0.0212 (6)	0.0088 (6)	0.0043 (4)	0.0164 (6)
N2A	0.0192 (4)	0.0139 (5)	0.0138 (4)	0.0027 (3)	0.0020 (3)	0.0009 (3)
C11A	0.0179 (5)	0.0141 (5)	0.0146 (5)	0.0019 (4)	0.0010 (4)	0.0013 (4)
C12A	0.0200 (5)	0.0203 (6)	0.0124 (5)	0.0010 (4)	0.0012 (4)	0.0017 (4)
C13A	0.0195 (5)	0.0186 (6)	0.0151 (5)	0.0032 (4)	0.0009 (4)	0.0034 (4)
S1B	0.01270 (10)	0.01178 (11)	0.00904 (10)	0.00008 (8)	0.00085 (8)	−0.00100 (8)
O1B	0.0207 (4)	0.0140 (4)	0.0151 (4)	−0.0021 (3)	0.0020 (3)	0.0028 (3)
O2B	0.0204 (4)	0.0147 (4)	0.0091 (3)	0.0010 (3)	−0.0011 (3)	−0.0004 (3)
O3B	0.0161 (4)	0.0197 (4)	0.0148 (4)	0.0036 (3)	0.0019 (3)	−0.0045 (3)
O4B	0.0345 (5)	0.0241 (5)	0.0217 (5)	−0.0021 (4)	0.0052 (4)	0.0054 (4)
C1B	0.0143 (4)	0.0160 (5)	0.0192 (5)	−0.0007 (4)	0.0007 (4)	−0.0069 (4)
C2B	0.0132 (4)	0.0127 (5)	0.0164 (5)	−0.0005 (3)	0.0020 (4)	−0.0034 (4)
C3B	0.0216 (5)	0.0129 (5)	0.0213 (6)	−0.0010 (4)	0.0023 (4)	−0.0001 (4)
C4B	0.0219 (6)	0.0204 (6)	0.0277 (7)	0.0068 (5)	0.0021 (5)	0.0034 (5)
C5B	0.0154 (5)	0.0174 (6)	0.0230 (6)	0.0023 (4)	0.0050 (4)	−0.0003 (4)
C6B	0.0206 (5)	0.0207 (6)	0.0239 (6)	0.0017 (4)	0.0057 (5)	−0.0081 (5)
C7B	0.0170 (5)	0.0200 (6)	0.0227 (6)	−0.0009 (4)	0.0038 (4)	−0.0099 (5)
C8B	0.0155 (4)	0.0143 (5)	0.0165 (5)	−0.0011 (4)	0.0031 (4)	0.0005 (4)
C9B	0.0316 (7)	0.0209 (7)	0.0208 (6)	0.0027 (5)	0.0048 (5)	0.0050 (5)
C10B	0.0185 (5)	0.0195 (6)	0.0243 (6)	−0.0061 (4)	0.0019 (4)	−0.0039 (5)
N1B	0.0142 (4)	0.0156 (5)	0.0114 (4)	0.0014 (3)	−0.0004 (3)	0.0017 (3)
N2B	0.0139 (4)	0.0138 (5)	0.0214 (5)	0.0016 (3)	0.0000 (4)	0.0036 (4)
C11B	0.0145 (5)	0.0149 (5)	0.0165 (5)	0.0000 (4)	0.0026 (4)	−0.0016 (4)
C12B	0.0167 (5)	0.0162 (5)	0.0132 (5)	0.0013 (4)	0.0020 (4)	−0.0015 (4)
C13B	0.0184 (5)	0.0193 (6)	0.0136 (5)	−0.0026 (4)	−0.0007 (4)	0.0029 (4)

### Geometric parameters (Å, °)

**Table:**

S1A—O2A	1.4542 (10)	C11A—H11A	0.9300
S1A—O1A	1.4645 (9)	C12A—C13A	1.3566 (18)
S1A—O3A	1.4663 (8)	C12A—H12A	0.9300
S1A—C1A	1.8074 (12)	C13A—H13A	0.9300
O4A—C3A	1.2090 (15)	S1B—O3B	1.4560 (9)
C1A—C2A	1.5102 (17)	S1B—O2B	1.4622 (8)
C1A—H1AA	0.9700	S1B—O1B	1.4661 (9)
C1A—H1AB	0.9700	S1B—C1B	1.7822 (12)
C2A—C3A	1.5185 (16)	O4B—C3B	1.2088 (17)
C2A—C7A	1.563 (2)	C1B—C2B	1.5264 (16)
C2A—C8A	1.5678 (17)	C1B—H1BA	0.9700
C3A—C4A	1.5248 (17)	C1B—H1BB	0.9700
C4A—C5A	1.529 (2)	C2B—C3B	1.5297 (18)
C4A—H4AA	0.9700	C2B—C7B	1.5606 (18)
C4A—H4AB	0.9700	C2B—C8B	1.5663 (16)
C5A—C6A	1.534 (2)	C3B—C4B	1.5280 (19)
C5A—C8A	1.547 (2)	C4B—C5B	1.532 (2)
C5A—H5AA	0.9800	C4B—H4BA	0.9700
C6A—C7A	1.555 (2)	C4B—H4BB	0.9700
C6A—H6AA	0.9700	C5B—C6B	1.532 (2)
C6A—H6AB	0.9700	C5B—C8B	1.5499 (18)
C7A—H7AA	0.9700	C5B—H5BA	0.9800
C7A—H7AB	0.9700	C6B—C7B	1.5593 (18)
C8A—C10C	1.352 (5)	C6B—H6BA	0.9700
C8A—C9A	1.506 (3)	C6B—H6BB	0.9700
C8A—C9C	1.594 (3)	C7B—H7BA	0.9700
C8A—C10A	1.681 (4)	C7B—H7BB	0.9700
C9A—H9AA	0.9600	C8B—C9B	1.5347 (19)
C9A—H9AB	0.9600	C8B—C10B	1.5374 (18)
C9A—H9AC	0.9600	C9B—H9BA	0.9600
C9C—H9CA	0.9600	C9B—H9BB	0.9600
C9C—H9CB	0.9600	C9B—H9BC	0.9600
C9C—H9CC	0.9600	C10B—H10G	0.9600
C10A—H10A	0.9600	C10B—H10H	0.9600
C10A—H10B	0.9600	C10B—H10I	0.9600
C10A—H10C	0.9600	N1B—C11B	1.3311 (16)
C10C—H10D	0.9600	N1B—C12B	1.3803 (15)
C10C—H10E	0.9600	N1B—H1BC	0.8600
C10C—H10F	0.9600	N2B—C11B	1.3291 (16)
N1A—C11A	1.3297 (16)	N2B—C13B	1.3747 (17)
N1A—C12A	1.3786 (17)	N2B—H2BA	0.8600
N1A—H1AC	0.8600	C11B—H11B	0.9300
N2A—C11A	1.3327 (16)	C12B—C13B	1.3552 (18)
N2A—C13A	1.3793 (16)	C12B—H12B	0.9300
N2A—H2AA	0.8600	C13B—H13B	0.9300
			
O2A—S1A—O1A	113.02 (6)	C13A—N2A—H2AA	125.5
O2A—S1A—O3A	112.24 (6)	N1A—C11A—N2A	108.26 (11)
O1A—S1A—O3A	111.57 (5)	N1A—C11A—H11A	125.9
O2A—S1A—C1A	106.46 (7)	N2A—C11A—H11A	125.9
O1A—S1A—C1A	108.37 (6)	C13A—C12A—N1A	107.11 (11)
O3A—S1A—C1A	104.61 (6)	C13A—C12A—H12A	126.4
C2A—C1A—S1A	119.53 (9)	N1A—C12A—H12A	126.4
C2A—C1A—H1AA	107.4	C12A—C13A—N2A	106.65 (11)
S1A—C1A—H1AA	107.4	C12A—C13A—H13A	126.7
C2A—C1A—H1AB	107.4	N2A—C13A—H13A	126.7
S1A—C1A—H1AB	107.4	O3B—S1B—O2B	112.27 (5)
H1AA—C1A—H1AB	107.0	O3B—S1B—O1B	113.28 (6)
C1A—C2A—C3A	118.10 (11)	O2B—S1B—O1B	111.96 (5)
C1A—C2A—C7A	116.16 (11)	O3B—S1B—C1B	104.81 (5)
C3A—C2A—C7A	102.95 (11)	O2B—S1B—C1B	106.33 (5)
C1A—C2A—C8A	115.32 (11)	O1B—S1B—C1B	107.57 (6)
C3A—C2A—C8A	99.42 (9)	C2B—C1B—S1B	118.47 (8)
C7A—C2A—C8A	102.31 (12)	C2B—C1B—H1BA	107.7
O4A—C3A—C2A	127.55 (11)	S1B—C1B—H1BA	107.7
O4A—C3A—C4A	125.97 (11)	C2B—C1B—H1BB	107.7
C2A—C3A—C4A	106.46 (10)	S1B—C1B—H1BB	107.7
C3A—C4A—C5A	102.31 (10)	H1BA—C1B—H1BB	107.1
C3A—C4A—H4AA	111.3	C1B—C2B—C3B	113.79 (10)
C5A—C4A—H4AA	111.3	C1B—C2B—C7B	119.69 (10)
C3A—C4A—H4AB	111.3	C3B—C2B—C7B	103.70 (10)
C5A—C4A—H4AB	111.3	C1B—C2B—C8B	115.55 (10)
H4AA—C4A—H4AB	109.2	C3B—C2B—C8B	99.72 (9)
C4A—C5A—C6A	106.96 (14)	C7B—C2B—C8B	101.75 (9)
C4A—C5A—C8A	101.83 (11)	O4B—C3B—C4B	126.53 (13)
C6A—C5A—C8A	103.31 (13)	O4B—C3B—C2B	126.68 (12)
C4A—C5A—H5AA	114.5	C4B—C3B—C2B	106.78 (10)
C6A—C5A—H5AA	114.5	C3B—C4B—C5B	101.81 (10)
C8A—C5A—H5AA	114.5	C3B—C4B—H4BA	111.4
C5A—C6A—C7A	102.63 (13)	C5B—C4B—H4BA	111.4
C5A—C6A—H6AA	111.2	C3B—C4B—H4BB	111.4
C7A—C6A—H6AA	111.2	C5B—C4B—H4BB	111.4
C5A—C6A—H6AB	111.2	H4BA—C4B—H4BB	109.3
C7A—C6A—H6AB	111.2	C6B—C5B—C4B	106.29 (12)
H6AA—C6A—H6AB	109.2	C6B—C5B—C8B	102.98 (10)
C6A—C7A—C2A	104.38 (13)	C4B—C5B—C8B	102.65 (10)
C6A—C7A—H7AA	110.9	C6B—C5B—H5BA	114.5
C2A—C7A—H7AA	110.9	C4B—C5B—H5BA	114.5
C6A—C7A—H7AB	110.9	C8B—C5B—H5BA	114.5
C2A—C7A—H7AB	110.9	C5B—C6B—C7B	103.23 (10)
H7AA—C7A—H7AB	108.9	C5B—C6B—H6BA	111.1
C10C—C8A—C9A	91.9 (2)	C7B—C6B—H6BA	111.1
C10C—C8A—C5A	115.18 (19)	C5B—C6B—H6BB	111.1
C9A—C8A—C5A	121.34 (19)	C7B—C6B—H6BB	111.1
C10C—C8A—C2A	118.6 (2)	H6BA—C6B—H6BB	109.1
C9A—C8A—C2A	117.82 (16)	C6B—C7B—C2B	103.83 (10)
C5A—C8A—C2A	94.20 (11)	C6B—C7B—H7BA	111.0
C10C—C8A—C9C	111.1 (2)	C2B—C7B—H7BA	111.0
C9A—C8A—C9C	19.89 (13)	C6B—C7B—H7BB	111.0
C5A—C8A—C9C	106.14 (19)	C2B—C7B—H7BB	111.0
C2A—C8A—C9C	110.03 (16)	H7BA—C7B—H7BB	109.0
C10C—C8A—C10A	11.72 (18)	C9B—C8B—C10B	108.40 (11)
C9A—C8A—C10A	103.2 (2)	C9B—C8B—C5B	113.06 (11)
C5A—C8A—C10A	110.47 (13)	C10B—C8B—C5B	114.08 (10)
C2A—C8A—C10A	109.62 (14)	C9B—C8B—C2B	114.39 (10)
C9C—C8A—C10A	122.6 (2)	C10B—C8B—C2B	112.29 (10)
C8A—C9A—H9AA	109.5	C5B—C8B—C2B	94.24 (9)
C8A—C9A—H9AB	109.5	C8B—C9B—H9BA	109.5
H9AA—C9A—H9AB	109.5	C8B—C9B—H9BB	109.5
C8A—C9A—H9AC	109.5	H9BA—C9B—H9BB	109.5
H9AA—C9A—H9AC	109.5	C8B—C9B—H9BC	109.5
H9AB—C9A—H9AC	109.5	H9BA—C9B—H9BC	109.5
C8A—C9C—H9CA	109.5	H9BB—C9B—H9BC	109.5
C8A—C9C—H9CB	109.5	C8B—C10B—H10G	109.5
H9CA—C9C—H9CB	109.5	C8B—C10B—H10H	109.5
C8A—C9C—H9CC	109.5	H10G—C10B—H10H	109.5
H9CA—C9C—H9CC	109.5	C8B—C10B—H10I	109.5
H9CB—C9C—H9CC	109.5	H10G—C10B—H10I	109.5
C8A—C10A—H10A	109.5	H10H—C10B—H10I	109.5
C8A—C10A—H10B	109.5	C11B—N1B—C12B	109.21 (10)
H10A—C10A—H10B	109.5	C11B—N1B—H1BC	125.4
C8A—C10A—H10C	109.5	C12B—N1B—H1BC	125.4
H10A—C10A—H10C	109.5	C11B—N2B—C13B	109.28 (11)
H10B—C10A—H10C	109.5	C11B—N2B—H2BA	125.4
C8A—C10C—H10D	109.5	C13B—N2B—H2BA	125.4
C8A—C10C—H10E	109.5	N2B—C11B—N1B	107.93 (11)
H10D—C10C—H10E	109.5	N2B—C11B—H11B	126.0
C8A—C10C—H10F	109.5	N1B—C11B—H11B	126.0
H10D—C10C—H10F	109.5	C13B—C12B—N1B	106.57 (11)
H10E—C10C—H10F	109.5	C13B—C12B—H12B	126.7
C11A—N1A—C12A	108.91 (11)	N1B—C12B—H12B	126.7
C11A—N1A—H1AC	125.5	C12B—C13B—N2B	107.01 (11)
C12A—N1A—H1AC	125.5	C12B—C13B—H13B	126.5
C11A—N2A—C13A	109.07 (11)	N2B—C13B—H13B	126.5
C11A—N2A—H2AA	125.5		
			
O2A—S1A—C1A—C2A	−105.42 (12)	C12A—N1A—C11A—N2A	0.11 (14)
O1A—S1A—C1A—C2A	16.45 (14)	C13A—N2A—C11A—N1A	−0.31 (14)
O3A—S1A—C1A—C2A	135.58 (11)	C11A—N1A—C12A—C13A	0.14 (15)
S1A—C1A—C2A—C3A	62.08 (16)	N1A—C12A—C13A—N2A	−0.32 (15)
S1A—C1A—C2A—C7A	−60.97 (16)	C11A—N2A—C13A—C12A	0.40 (15)
S1A—C1A—C2A—C8A	179.34 (11)	O3B—S1B—C1B—C2B	−176.85 (9)
C1A—C2A—C3A—O4A	−18.12 (19)	O2B—S1B—C1B—C2B	64.08 (11)
C7A—C2A—C3A—O4A	111.36 (15)	O1B—S1B—C1B—C2B	−56.02 (11)
C8A—C2A—C3A—O4A	−143.57 (14)	S1B—C1B—C2B—C3B	101.52 (11)
C1A—C2A—C3A—C4A	160.54 (11)	S1B—C1B—C2B—C7B	−21.79 (16)
C7A—C2A—C3A—C4A	−69.99 (12)	S1B—C1B—C2B—C8B	−143.94 (9)
C8A—C2A—C3A—C4A	35.08 (13)	C1B—C2B—C3B—O4B	−20.75 (19)
O4A—C3A—C4A—C5A	178.27 (13)	C7B—C2B—C3B—O4B	110.90 (15)
C2A—C3A—C4A—C5A	−0.41 (13)	C8B—C2B—C3B—O4B	−144.36 (14)
C3A—C4A—C5A—C6A	72.78 (14)	C1B—C2B—C3B—C4B	157.98 (11)
C3A—C4A—C5A—C8A	−35.27 (14)	C7B—C2B—C3B—C4B	−70.38 (12)
C4A—C5A—C6A—C7A	−68.92 (19)	C8B—C2B—C3B—C4B	34.36 (12)
C8A—C5A—C6A—C7A	38.09 (18)	O4B—C3B—C4B—C5B	178.76 (14)
C5A—C6A—C7A—C2A	−4.3 (2)	C2B—C3B—C4B—C5B	0.04 (14)
C1A—C2A—C7A—C6A	−156.62 (14)	C3B—C4B—C5B—C6B	72.54 (13)
C3A—C2A—C7A—C6A	72.72 (16)	C3B—C4B—C5B—C8B	−35.24 (13)
C8A—C2A—C7A—C6A	−30.11 (17)	C4B—C5B—C6B—C7B	−71.47 (13)
C4A—C5A—C8A—C10C	−68.8 (2)	C8B—C5B—C6B—C7B	36.08 (13)
C6A—C5A—C8A—C10C	−179.6 (2)	C5B—C6B—C7B—C2B	−1.55 (14)
C4A—C5A—C8A—C9A	−178.0 (2)	C1B—C2B—C7B—C6B	−161.59 (11)
C6A—C5A—C8A—C9A	71.1 (2)	C3B—C2B—C7B—C6B	70.33 (12)
C4A—C5A—C8A—C2A	55.64 (13)	C8B—C2B—C7B—C6B	−32.87 (13)
C6A—C5A—C8A—C2A	−55.21 (14)	C6B—C5B—C8B—C9B	63.83 (13)
C4A—C5A—C8A—C9C	167.90 (17)	C4B—C5B—C8B—C9B	174.13 (11)
C6A—C5A—C8A—C9C	57.05 (19)	C6B—C5B—C8B—C10B	−171.69 (11)
C4A—C5A—C8A—C10A	−57.12 (16)	C4B—C5B—C8B—C10B	−61.40 (13)
C6A—C5A—C8A—C10A	−167.96 (15)	C6B—C5B—C8B—C2B	−54.96 (11)
C1A—C2A—C8A—C10C	−60.1 (2)	C4B—C5B—C8B—C2B	55.33 (11)
C3A—C2A—C8A—C10C	67.3 (2)	C1B—C2B—C8B—C9B	66.55 (14)
C7A—C2A—C8A—C10C	172.9 (2)	C3B—C2B—C8B—C9B	−171.07 (11)
C1A—C2A—C8A—C9A	49.3 (3)	C7B—C2B—C8B—C9B	−64.75 (13)
C3A—C2A—C8A—C9A	176.6 (2)	C1B—C2B—C8B—C10B	−57.54 (14)
C7A—C2A—C8A—C9A	−77.8 (3)	C3B—C2B—C8B—C10B	64.84 (12)
C1A—C2A—C8A—C5A	178.22 (12)	C7B—C2B—C8B—C10B	171.16 (11)
C3A—C2A—C8A—C5A	−54.43 (12)	C1B—C2B—C8B—C5B	−175.74 (10)
C7A—C2A—C8A—C5A	51.17 (13)	C3B—C2B—C8B—C5B	−53.37 (10)
C1A—C2A—C8A—C9C	69.4 (2)	C7B—C2B—C8B—C5B	52.95 (11)
C3A—C2A—C8A—C9C	−163.3 (2)	C13B—N2B—C11B—N1B	0.55 (14)
C7A—C2A—C8A—C9C	−57.7 (2)	C12B—N1B—C11B—N2B	−0.35 (14)
C1A—C2A—C8A—C10A	−68.30 (16)	C11B—N1B—C12B—C13B	0.01 (14)
C3A—C2A—C8A—C10A	59.06 (15)	N1B—C12B—C13B—N2B	0.32 (13)
C7A—C2A—C8A—C10A	164.65 (14)	C11B—N2B—C13B—C12B	−0.54 (14)

### Hydrogen-bond geometry (Å, °)

**Table:**

*D*—H···*A*	*D*—H	H···*A*	*D*···*A*	*D*—H···*A*
N1A—H1AC···O2B^i^	0.86	2.46	2.9479 (14)	116
N1A—H1AC···O3A^ii^	0.86	2.00	2.8293 (14)	161
N2A—H2AA···O1B^iii^	0.86	1.89	2.7235 (14)	164
N1B—H1BC···O1A^i^	0.86	1.88	2.7231 (14)	165
N2B—H2BA···O2B^iii^	0.86	1.97	2.7412 (14)	148
N2B—H2BA···O3A^iv^	0.86	2.43	3.0111 (14)	125
C7A—H7AB···O1A	0.97	2.53	3.0257 (19)	111
C11A—H11A···O2B^i^	0.93	2.49	2.9716 (16)	113
C11A—H11A···O3B^i^	0.93	2.35	3.2648 (15)	170
C11B—H11B···O2A^iv^	0.93	2.33	3.2103 (16)	159
C9A—H9AC···O2A^i^	0.96	2.59	3.469 (3)	152
C12B—H12B···O3B^i^	0.93	2.39	2.9950 (15)	122
C12B—H12B···O4A^i^	0.93	2.49	3.0155 (16)	116
C13A—H13A···O2A^iii^	0.93	2.44	3.0167 (16)	120
C13A—H13A···O4A^iii^	0.93	2.39	3.2591 (17)	155
C13B—H13B···O1B^iii^	0.93	2.58	3.2720 (16)	131
C7B—H7BA···O2B	0.97	2.47	3.0613 (16)	119
C5A—H5AA···Cg1	0.98	2.83	3.5459 (5)	131

Symmetry codes: (i) *x*−1, *y*, *z*; (ii) *x*−1, *y*, *z*+1; (iii) −*x*+2, *y*−1/2, −*z*+1; (iv) −*x*+2, *y*−1/2, −*z*.
